# Brushing Effect on the Properties of Glass Ionomer Cement Modified by Hydroxyapatite Nanoparticles or by Bioactive Glasses

**DOI:** 10.1155/2022/1641041

**Published:** 2022-02-21

**Authors:** Rafael A. Martins, Luana M. Marti, Ana C. B. Mendes, Camila Fragelli, Mario Cilense, Angela C. C. Zuanon

**Affiliations:** ^1^Department of Morphology and Pediatric Dentistry, São Paulo State University-UNESP, School of Dentistry, Mail Box: 331, 1680 Humaitá St., Araraquara, São Paulo 14801-903, Brazil; ^2^Department of Chemical Physics, São Paulo State University-UNESP, School of Dentistry, 55 Prof. Francisco Degni St., Araraquara, São Paulo 14800-060, Brazil

## Abstract

This study evaluated the physical and mechanical properties of glass ionomer cement (GIC) associated with 5% hydroxyapatite nanoparticles (NPHAps) and 10% bioactive glass (BAG) 45S5 before and after brushing at different storage times. Surface roughness was evaluated using a rugosimeter, Vickers hardness using a microdurometer, and mass variation measured in an analytical balance at 1, 7, 15, 30, and 60 days before and after the brushing test, with the aid of toothbrushing simulator and soft bristle toothbrushes. Nonnormal distribution was observed, and the nonparametric Wilcoxon and Kruskal–Wallis tests followed by Dunn's were performed, with a significance level of 5%. We observed higher values for mass loss on the first day for all groups. The surface roughness was lower in the control and NP groups, 30 days after brushing. Higher values for hardness were found in the control group and lower ones for NP, after brushing. The control and BAG groups presented a decrease in hardness over time. The NP group presented the highest values before brushing, while the control group had the highest values after brushing. The association of NPHPa with the GIC is the most promising combination, since it presented satisfactory values for surface hardness. However, conventional GIC not associated with NPHPa or BAG is still an option, since it is available in the market and the most economically viable option.

## 1. Introduction

The glass ionomer cement (GIC) has an ionic exchange mechanism with dental structures, which allows chemical adhesion to both enamel and dentin [1]. In addition, it presents characteristics such as biocompatibility, ability to release and reincorporate fluoride from the oral environment and linear thermal expansion coefficient similar to dentin [2, 3]. However, GIC has limitations due to mechanical properties, such as low wear resistance, hardness, and tensile and compressive diametral strength [4].

It is capable of inducing the remineralization of dentin and enamel, since the fluoride release assists in the formation of fluorapatite, besides being considered the material of choice for performing atraumatic restorative treatment, as it has confirmed caries control action [5, 6].

Takahashi et al. [7] and Palmer et al. [8] associated the GIC to chlorhexidine in order to potentiate the antibacterial property of the material. These researchers found an increase in antibacterial property compared to conventional GIC, considering possible changes in the physical and mechanical properties of the material due to the modification of its original composition.

Incorporation of nanoparticles (NPs) into various restorative materials in order to improve their mechanical [9, 10], physical, and antibacterial properties [4, 11] is also widely evaluated in literature. NPs have extremely reduced size, which results in a large superficial area and higher contact with the environment in which they are found [12]. They increase the permeability of cell membranes, as well as the flow out of the cytoplasmic contents of the cell, facilitating their penetration into the microorganism, leading to the destruction of the lipids and cellular proteins of the cell [12–14].

The hydroxyapatite [HAp: Ca_10_(PO_4_)_6_OH_2_] demonstrates biocompatibility, compositions and structure of apatite-like crystals, which is present in dental structures in humans and in bone tissues. The NP of hydroxyapatite (NPHAp), when added to the restorative GIC, demonstrated an increase in flexural strength and in the release of fluoride ions, acting not only as a reinforcement of the material but also as an adsorbent component and an ion exchange agent, resulting in better chemical and mechanical properties [15]. Alatawi et al. [16] found an antibacterial increase with the association of GIC and NPHAp due to fluoride ions release.

Moshaverinia et al. [4] also demonstrated that the incorporation of NPHAp into the GIC provided improvements in compressive strength and tensile diametral strength. Recent studies have also shown that the association of GIC with NP can reduce the number of pores and increase compressive strength. Association with NPHAp may also increase flexural and shear bond strength [17, 18]. Kantovitz et al. [19] found greater compressive strength when he combined GIC with titanium NP and also less mass loss with no difference in roughness after brushing.

We have also studied the addition of bioactive glasses (BAG) to the GIC for the improvement of its remineralizing properties, which have been explored in the literature [20–22]. In dentistry, there are several applications of BAG, such as implantology, maxillofacial surgery, periodontics, pulp therapy, and restorative materials. BAGs have the ability to chemically bind to bone minerals. They are composed of oxides of calcium, phosphorus, silicon, and sodium in different proportions which precipitate and confer remineralizing action when in contact with dentin [20, 21]. According to Bakry et al. [22], BAGs have the capacity to penetrate the dentinal tubules and the presence of calcium and phosphate may be capable of remineralizing the subsurface demineralized enamel.

BAGs, when in aqueous environments, form an apatite layer on its surface, both in vitro and in vivo [23]. Not only fluoride ions but also calcium and phosphate ions are released from the GIC when the material was associated with BAG [24]. Its high pH value when in aqueous medium [25, 26] can make changes in the mechanical properties of the material, such as reduction of surface hardness or increase of flexural strength [27].

GIC's performance associated with NP or BAG depends on the maintenance of the original characteristics of these materials and the quality of their surface, which has a primary role to be in contact with the oral environment and its elements. Once there is an increase in surface roughness, colonization of microorganisms becomes easier and faster [28]. The wear of the material also results in increased roughness, which also occurs due to dental brushing and dentifrice quality and toothbrush quality and pressure exerted on it, besides brushing frequency [29]. Inherent factors of the material such as integrity between the matrix and the glass particles, size, shape of the particles, and porosity should also be considered [30].

Many studies demonstrate the remineralizing and antibacterial capacity of BAG and NP, respectively, when associated with different restorative materials [4, 15, 20, 26]. It is important to develop researches that answer the doubts about the possible changes in physical, chemical, and mechanical properties when BAG and NP are associated with GIC, in addition to changes that may occur due to brushing.

Considering that GIC associated with 5% of NPHAp and 10% of BAG 45S5 lead to the improvement of its properties, this study evaluated the surface roughness, Vickers hardness, and mass variation of, before, and after brushing at different storage times.

## 2. Materials and Methods

This is an experimental laboratory.

The test specimens were made with GIC restorative (Ketac Molar EasyMix-3M ESPE, Campinas, SP, Brazil) and divided into 3 experimental groups, with 50 specimens in each.

Ten percent (10%) BAG 45S5 [21, 26] and 5% NPHAp [4] (SIGMA-ALDRICH; ref: 677418-10; batch: MKBW9108V) were added to the GIC.

The amount of powder used for the Control group was established using the arithmetic mean. From this measurement, the desired weight percentage of the GIC powder was removed and the same percentage of NPHAp or BAG was added. After homogenization, the powder was agglutinated with a drop of the liquid. This drop was dispensed onto the mixing pad with the bottle positioned vertically as indicated by the manufacturer (Powder/liquid ratio 2 : 1).

With the use of a Centrix syringe (DFL and Comércio S.A. Rio de Janeiro, RJ, Brazil), the GIC associated with NPHAp or BAG was inserted in silicone matrices with 3 mm height and 6 mm diameter [31]. For complete setting reaction of the material, the specimens were stored in a suitable container with approximately 100% relative air humidity at 37°C incubator for 24 hours [32]. Right after, the specimens were submitted to tests of mass variation, Vickers hardness, and surface roughness, before and after brushing test for different periods of time.

After the first 24 hours, the specimens were weighed daily by means of an analytical balance (Ind. Com. Eletro-Eletrônica GE-HA-KA Ltda, model BG 440, São Paulo, Brazil), once a day, until the initial mass (IM) was stabilized, and the IM value was obtained. After the brushing, a new weighing sequence was performed to determine the final mass (FM). During all experimental times, the specimens were kept immersed in deionized water for 1, 7, 15, 30, and 60 days. As the measurements were obtained every 24 hours, the specimen mass was considered to be stable from the moment that five consecutive measurements were observed with the same value. And the mass variation values were obtained based on the difference between the initial mass (before brushing) and the final mass (after brushing) [28].

The surface roughness of the specimens with a cut-off of 0.25 mm was analyzed, and the values (Ra) were obtained by arithmetic mean between the peaks and valleys recorded by the rugosimeter (Surfcorder SE 1700, Kosaka Laboratory Ltd., Kosaka, Japan). On each surface, three readings were made in different positions, starting 2 mm below the edge of the specimen, always passing through its center.

The Vickers hardness reading was performed by a single operator in a digital microdriometer (Micromet 2100-Buehler Ltda., Lake Bluff, Illinois, USA), applying a load of 50 kgf for 30 seconds on the surface of the specimens. In each specimen, six indentations were made at equidistant points. The results expressed Vickers hardness values (VHN) directly by the test machine.

**Figure 1:**
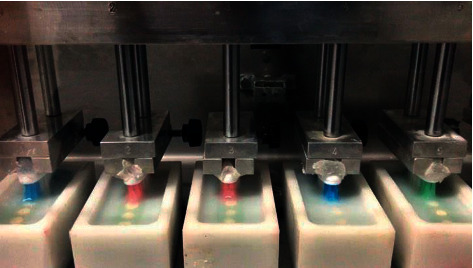
Plastic tubs with the dies accommodated, fitted to the brushing machine.

The brushing test was performed in a brushing simulation machine (MEV-2T-Odeme Dental Research, Miami, USA) with a linear course of 60 mm extension in 2 seconds (30,000 cycles simulating 3 years of brushing) [33], with the aid of soft bristle toothbrushes (Dental PowerDent Classic Power Brush, PowerDent, São Paulo, Brazil) and 6 g of toothpaste “Colgate Máxima Proteção Anticáries” (Colgate, 90 grams with 1450 ppm fluorine-Colgate-Palmolive Industrial LTDA, São Paulo, Brazil) mixed with 6 ml of water (Figure 1) [34].

The data obtained were statistically analyzed using the statistical package SPSS 22.0 (SPSS Inc., Chicago, IL, USA), for normality using the Kolmogorov–Smirnov test where a nonnormal distribution was observed. For the analysis of superficial roughness and Vickers hardness comparing values before and after brushing for each time interval, in each experimental group, Wilcoxon's nonparametric test was performed. For the analysis of surface roughness and Vickers hardness over time, divided before and after the brushing test, in each experimental group and for comparison between groups at the various times, the Kruskal–Wallis nonparametric test was performed, followed by the Dunn test. The nonparametric Kruskal–Wallis test was followed by the Dunn test using subtraction of the IM of the test specimens, weighed before immersion, and the FM of each specimen after the tests. All were performed with a significance level of 5%.

## 3. Results

The mass variation showed a statistically significant difference when considering each experimental group separately (Table 1). In Control and BAG groups, significant mass loss was observed on the first day of experiment (*p* = 0.016). For the NPHAp group mass loss was observed until the seventh day (*p* **≤** 0.001).

There was still a statistical difference between the groups at 1, 30, and 60 days. On the first day, this difference was representative between groups NPHAp and BAG, with greater loss of mass for the BAG (*p* = 0.016). At 30 days, the BAG group showed greater statistically significant mass loss compared to the control and NPHAp (*p* **≤** 0.001). At 60 days, the NPHAp group presented higher mass, being statistically different from the Control and BAG groups (*p* **≤** 0.001).

The surface roughness, before and after brushing test, showed a statistically significant difference for the time of 30 days, when the Control (*p* = 0.011) and NPHAp (*p* = 0.037) groups had the lowest roughness value after brushing test (Table 2).

Statistically significant difference was observed for surface roughness in the Control group only after brushing in the first and seventh days (*p* = 0.006). Higher values for surface roughness were observed for the 60^th^ day (Table 3).

It was observed over time, when considering the values between the experimental groups before brushing, that there was a statistically significant difference at 1 and 7 days. In the first day, higher roughness value was presented by the BAG group (*p* = 0.006). At 7 days, both NPHAp and BAG groups had higher surface roughness values (*p* = 0.004). After the brushing test at 1 and 7 days, they were also the ones that presented statistical difference, and at 1 day the Control group presented lower surface roughness (*p* = 0.003) and at 7 days, the Control group presented lower roughness when compared to the BAG group (*p* = 0.003) (Table 3).

The Control group presented increase in Vickers hardness after brushing test for 1, 7, and 30 days (*p* = 0.007; *p* = 0.047; *p* = 0.008). The NPHAp group presented decrease only for the 7^th^ day, after the brushing test (*p* = 0.009). For the BAG group, no significant differences were found (Table 4).

When comparing over time (Table 5), it was observed that in the Control group there was a higher value of Vickers hardness for 7 days, before brushing (*p* = 0.010). For the BAG group, the highest value of Vickers hardness was in the first day of, before (*p* = 0.002), and after brushing (*p* = 0.009) (Table 5).

Higher values of Vickers hardness with statistical difference over time between the different experimental groups, before brushing, were for the NPHAp group. After brushing, only at 30 days' control group presented the highest values of Vickers hardness (*p* = 0.031).

**Figure 2:**
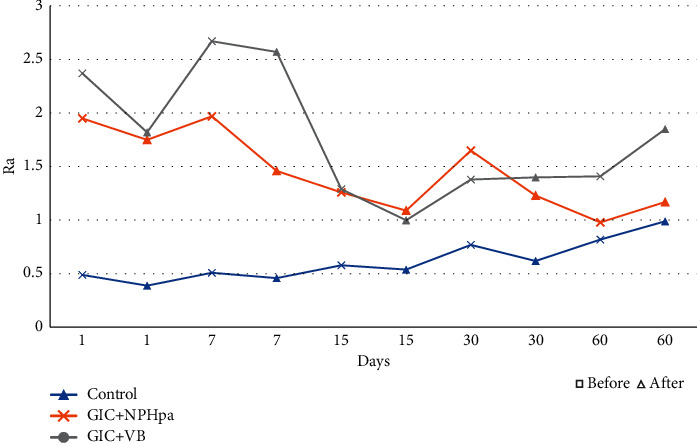
Variability of surface roughness (Ra) before and after brushing over time (days).

**Figure 3:**
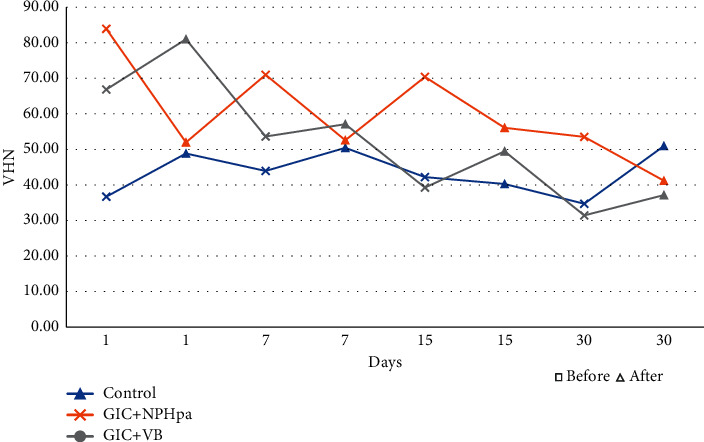
Variability of Vickers hardness (VHN) before and after brushing over time (days).

And for a comprehensive view of the data over time, the following images show the pre- and postbrushing variability of surface roughness (Figure 2) and Vickers hardness (Figure 3).

## 4. Discussion

The use of BAG or NP has been studied [4, 15, 20, 26] in order to improve the remineralization and antibacterial activity of dental materials, without, however, changing their physical or mechanical properties. Similar to the literature [4, 11, 21, 27, 30, 34, 35], our study found statistically significant differences in these properties of the GIC Ketac Molar EasyMix when associated with BAG or NP.

The mass loss, which indicates the amount of material wear [27, 36], is a property that, when altered, can cause serious damage to the longevity of the restoration. Factors such as acid base reaction of the GIC, presence of air bubbles in their interior, and proportion and size of the glass particles are related to the variation of this property, increasing its susceptibility to erosion, pronounced displacement of inorganic particles and greater exposure of air bubbles [29, 37]. The syneresis and/or imbibing of this material should also be considered [28, 38].

In this study, we observed greater mass loss in the Control and BAG groups on the first day compared to other days, and for the NPHAp group, in the first and seventh days. The first days are critical for the complete maturation of GIC [37], and the subjection of this material to the test may have led to greater changes on its surface, such as loss of glass particles and/or organic matrix, resulting in a lower mass and consequently higher wear of the material (Table 1).

When compared between the groups, the greatest mass loss was observed in the BAG group (Table 1), probably due to greater dissolution of the organic component of GIC, which has a great capacity for water absorption, resulting in poor binding between the BAG particles and the matrix of the GIC [21]. Statistically significant differences were found in the mass variation of some types of GIC [36], relating this fact to the difference in the amount of water inside the materials before their weighing.

Table 1 shows an increase in mass at times of 30 and 60 days for the Control group, 15 to 60 days for the NPHAp group, and 15 days for the BAG group, possibly due to fluoride recharging by the GIC, when in contact with the toothpaste during the brushing test. Panigrahi et al. [39] and Yli-Urpo et al. [21] observed that after the association of a GIC with the remineralizing material there was a higher release of fluoride, and consequently a higher incorporation of these ions.

The degradation of restorative materials may also be related to the pH decrease of the buccal cavity, sorption of water, and erosion of these materials, which results in the degradation of the matrix and interface of its surface and may also result in greater surface roughness. In addition to the accumulation of biofilm on the material surface, it also results in alterations in aesthetics, cracking, change in color, and reflection of light [2, 29] and consequent decrease in the longevity of restoration due to caries lesions, gingival inflammation, among others [38].

After being submitted to the brushing test, the Control and NP groups showed a decrease in surface roughness at 30 days (Table 2) probably due to the possible polishing of this surface. Bala et al. [40] evaluated the surface roughness of a nanoparticulate GIC in comparison to conventional GICs and found lower roughness values for the former, after polishing.

Although it presented the lowest values for surface roughness when compared to the other experimental groups, the control group demonstrated an increase of this property, directly proportional to the time (Table 3). In this work, Cibim et al. [34] evaluated the surface roughness of a modified GIC by TiO_2_ NP and found that, regardless of the concentration of NP, it did not affect the distribution and bonding between NP particles and the GIC matrix. They also reported that particle size affects surface roughness and that nanometric particles may favor this property.

Mitra et al. [35] pointed out the tendency to form clusters of NP when associated with a dental material, which, when subjected to abrasion caused by brushing, may have the surface clusters detached, leaving the surface of the restorative material with minor defects, resulting in better optical properties.

When incorporating BAG to a GIC, Valanezhad et al. [27] found cracks in the surface of the material, caused by the tensions generated during sample preparation and inadequate dispersion of the BAG particles within the GIC matrix. They reported that BAG particles represented centers of stress concentration, where fissures began. This report supports the data obtained in this study, which demonstrated the highest values of surface roughness for the BAG (Table 3). The authors also observed dissolution of the GIC matrix after immersion of the material in PBS, with increased surface roughness.

Thomassewski et al. [36] observed that all the GICs not associated with NP or BAG suffered wear after simulated brushing and increased roughness. In this study, it is also possible to observe increase of roughness for the control group after the brushing, directly proportional to the time of storage (Table 3).

The evaluation of the superficial hardness is also important when we consider the success of a restoration, considering that this property is altered by exposure to water and to saliva [41], besides the composition of the polyacrylic acid that makes up the GIC [42]. This study found a statistically significant increase in the values of this property in the Control group and decrease in the NPHAp group after the brushing test (Table 4). Analyzing each group separately over time, the Control and BAG groups showed a decrease in surface hardness values before the brushing test (Table 5).

The NP presented higher hardness values before the brushing test, and after the same test the Control Group presented the higher values compared to the BAG (Table 5). According to Xie et al. [30], the presence of dispersed glass particles in the polymer matrix can result in higher values of surface hardness.

Prentice et al. [11] suggested that the addition of NP to GIC results in less glass particles on the surface of the material, providing a more intense acid reaction and a decrease in its hardness. Panahandeh et al. [43] also found a decrease in Vickers hardness when GIC was associated with NP and the formation of clusters was pointed out as responsible for this. Moshaverinia et al. [4], however, observed an increase in surface hardness when GIC was associated with fluorapatite, corroborating the values obtained in this study, before the brushing test. Increased surface hardness values were also found by Moshaverinia et al. [37] after one week of storage in distilled water of a fluorapatite NP modified GIC. This is possibly due to the intensity increase of the acid-base reaction of the GIC due to low release of calcium ions from fluorapatite NP, with higher number of bridges with high phosphate and calcium ion concentration, which reinforced the matrix, improving the interaction between organic and inorganic networks. According to the authors as the cement ages in distilled water, it promotes more cross-linking, leading to increased surface hardness values.

Valanezhad et al. [27] found, as well as this study, a decrease in the values of this property, probably due to the presence of cracks in the material. In an aqueous environment, the GIC absorbs water resulting in poor bonding between the BAG particles and the GIC matrix, which leads to a decrease in the surface hardness of the material, as well as ions that precipitate on the glass particles [44]. Yli-Urpo et al. [21] observed that when immersed in deionized water, GIC associated with BAG presented decreases in hardness values. The dissolution and precipitation of components may alter the surface morphology of the material, thus leading to variations in its properties [21, 27, 44].

Another factor that may have affected not only hardness, but also surface roughness, as already mentioned, is the wear that occurs naturally over time, or as simulated in this study, by brushing. This wear due to brushing can alter the aesthetic and structural characteristics of dental materials, leading to notable effects on hardness [45]. Kyoizumi et al. [46], however, in their study concluded that there is an influence of brushing on material properties, especially regarding the variation of the bristles, whether softer or harder. However, more than brushing, wear changes come from the combination with the type of material, not just the brushes. They conclude that the hardness grades of toothbrushes have minor effects on abrasion and surface roughness of composite resins. More in-depth studies on wear are needed, as it is very complex, especially evaluating the microstructural part of the surface of materials, since there is still no standardization in the evaluation of this property [47].

The use of hydroxyapatite as a remineralization system is based on a biomimetic approach that aims to restore the tooth with the same substance that constitutes its hard tissues [48]. In the study by Butera et al. [49], it was possible to observe that the use of a dentifrice containing Zn-carbonate hydroxyapatite on composite resin in the oral environment increased the deposition of calcium and silicon indicating the presence of remineralizing activity, being also a mechanism that can collaborate for the prevention of secondary decay. The findings of this research together with those found in the literature indicate the potential that exists in the association and use of hydroxyapatite with restorative materials.

This study has as a limitation of being in vitro, and the use of only one GIC, which was used because it is still one of the gold standards in the literature and the material of choice in the pediatric clinic of the institution where the research was conducted. Standardization when simulating brushing is also a limitation, as it is susceptible to several factors (brush type, applied force, and time).

The immersion solution also has limitations. Artificial saliva is the first option when thinking about simulating the oral cavity, but considering other tests to be performed by this study group, deionized water was used. This can lead to differences in the results, mainly because artificial saliva contains significant amounts of calcium and phosphate that can influence the properties of the GIC [50]. However, studies that compared immersion in artificial saliva and deionized and/or distilled water did not observe significant differences in several properties such as compressive strength [50] and surface degradation of the material [51] and found the same pattern of fluoride release [52]. And as all groups in this study were immersed in the same solution, the results are subject to comparison and validation.

In addition, this study was carried out mediately, requiring evaluations and confirmations of findings in long-term studies since in the search for a dental material with better properties for clinical use, the analysis of its properties and composition is interesting, guaranteeing adequate antibacterial activity and greater longevity of the restorations, without suffering excessive wear. The association of GIC with NPs or with BAGs has been widely studied, and in this same study, we brought the comparison of a type of NP (NPHAp) and a type of BAG (45S5), which is not easily found in the literature.

## 5. Conclusion

It is concluded that the association of NP or BAG with the GIC generated changes in the properties studied, and the association of NPHAp with the GIC is the most promising one, since it presented satisfactory values for surface hardness. However, conventional GIC not associated with NPHAp or BAG is still the best option found in vitro; since it presented the best results, it is already in the market and is economically the most viable option.

This study is considered to be of great clinical relevance since this GIC is widely used in pediatric dentistry, and constant scientific investigations to improve its properties are important to ensure its long-term clinical success.

## Figures and Tables

**Table 1:** Mass variation (g) over time (days)-mean of mass lost after brushing (standard deviation).

	1 day	7 days	15 days	30 days	60 days	
Control	0.00445^a,AB^ (0.000617)	0.00064^b^ (0.001267)	0.00008^b^ (0.000355)	−0.00003^b,A^ (0.000418)	−0.00010^b,A^ (0.000533)	**p** **≤** **0.001^∗^**
NPHAp	0.00348^a,A^ (0.003099)	0.00055^a^ (0.001742)	−0.00054^ab^ (0.000628)	−0.00358^ab,A^ (0.013017)	−0.00205^b,B^ (0.000682)	**p** **≤** **0.001^∗^**
BAG	0.00524^a,B^ (0.000981)	0.00110^ab^ (0.004984)	−0.00051^b^ (0.008022)	0.00200^ab,B^ (0.001679)	0.00009^b,A^ (0.000796)	**p** **≤** **0.001^∗^**
	**p** **=** **0.016^∗^**	*p* = 0.067	*p* = 0.054	**p** **≤** **0.001^∗^**	**p** **≤** **0.001^∗^**	

*Note*. Averages in the columns accompanied by uppercase letters and in the lines accompanied by lowercase letters do not present significant difference by the Kruskal–Wallis test (*p* > 0.05) and Dunn post test (*p* > 0.05). **^∗^**Source: own elaboration.

**Table 2:** Surface roughness (Ra) before and after the brushing test for each time interval (days), for each experimental group-mean (standard deviation).

	1 day		7 days		15 days		30 days		60 days	
	BB	AB	BB	AB	BB	AB	BB	AB	BB	AB
Control	0.49 (0.38)	0.39 (0.29)	0.51 (0.29)	0.46 (0.23)	0.58 (0.28)	0.54 (0.26)	0.77 (0.30)	0.62 (0.20)	0.82 (0.40)	0.99 (0.41)
	*p* = 0.285		*p* = 0.508		*p* = 0.508		**p** **=** **0.011^∗^**		*p* = 0.221	

NPHAp	1.95 (1.36)	1.75 (1.21)	1.97 (0.92)	1.46 (0.92)	1.26 (1.10)	1.09 (0.82)	1.65 (1.48)	1.23 (1.06)	0.98 (0.99)	1.17 (0.98)
	*p* = 0.333		*p* = 0.059		*p* = 0.241		**p** **=** **0.037^∗^**		*p* = 0.508	

BAG	2.37 (1.29)	1.82 (0.59)	2.67 (1.76)	2.57 (1.87)	1.29 (0.85)	1.00 (0.80)	1.38 (0.96)	1.40 (0.88)	1.41 (1.28)	1.85 (1.83)
	*p* = 0.139		*p* = 0.959		*p* = 0.203		*p* = 0.799		*p* = 0.575	

*Note*. Averages with a statistically significant difference by the Wilcoxon test (*p* < 0.05). ^∗^Source: own elaboration.

**Table 3:** Surface roughness (Ra) over time (days), before and after brushing test, for each experimental group-mean (standard deviation).

	1 day	7 days	15 days	30 days	60 days	
	Before brushing					
Control	0.49^A^ (0.38)	0.51^A^ (0.29)	0.58 (0.28)	0.77 (0.30)	0.82 (0.40)	*p* = 0.099
NPHAp	1.95^AB^ (1.36)	1.97^B^ (0.92)	1.26 (1.10)	1.65 (1.48)	0.98 (0.99)	*p* = 0.440
BAG	2.37^B^ (1.29)	2.67^B^ (1.76)	1.29 (0.85)	1.38 (0.96)	1.41 (1.28)	*p* = 0.187
	**p** **=** **0.006^∗^**	**p** **=** **0.004∗**	*p* = 0.114	*p* = 0.632	*p* = 0.525	

	After brushing					
Control	0.39^a,A^ (0.29)	0.46^a,A^ (0.23)	0.54^ab^ (0.26)	0.62^ab^ (0.20)	0.99^b^ (0.41)	**p** **=** **0.006^∗^**
NPHAp	1.75^B^ (1.21)	1.46^AB^ (0.92)	1.09 (0.82)	1.23 (1.06)	1.17 (0.98)	*p* = 0.731
BAG	1.82^B^ (0.59)	2.57^B^ (1.87)	1.00 (0.80)	1.40 (0.88)	1.85 (1.83)	*p* = 0.073
	**p** **=** **0.003^∗^**	**p** **=** **0.003^∗^**	*p* = 0.238	*p* = 0.109	*p* = 0.506	

*Note*. Averages in the columns accompanied by uppercase letters and in the lines accompanied by lowercase letters do not present significant difference by the Kruskal–Wallis test (*p* > 0.05) and Dunn post test (*p* > 0.05). **^∗^**Source: own elaboration.

**Table 4:** Vickers hardness (MPa) before and after brushing for each time interval (days), for each experimental group-mean (standard deviation).

	1 day		7 days		15 days		30 days	
	BB	AB	BB	AB	BB	AB	BB	AB
Control	36.70 (7.00)	48.85 (7.62)	43.94 (6.26)	50.44 (10.22)	42.21 (8.04)	40.28 (9.54)	34.70 (5.72)	50.96 (12.98)
	**p** **=** **0.007^∗^**		**p** **=** **0.047^∗^**		*p* = 0.241		**p** **=** **0.008^∗^**	

NPHAp	83.92 (36.90)	51.97 (22.34)	70.99 (31.45)	52.58 (17.69)	70.40 (26.62)	56.08 (24.54)	53.52 (30.52)	41.19 (11.04)
	*p* = 0.074		**p** **=** **0.009^∗^**		*p* = 0.093		*p* = 0.386	

BAG	66.86 (23.68)	80.99 (37.97)	53.63 (18.22)	57.09 (22.70)	39.27 (14.80)	49.46 (15.40)	31.43 (12.57)	37.15 (9.85)
	*p* = 0.575		*p* = 0.799		*p* = 0.241		*p* = 0.169	

*Note*. Averages with a statistically significant difference by the Wilcoxon test (*p* < 0.05). ^∗^Source: own elaboration.

**Table 5:** Analysis of Vickers hardness over time, divided before and after brushing, in each group studied-means in VHN (standard deviation).

	1 day	7 days	15 days	30 days	
	Before brushing				
Control	36.70^ab,A^ (7.00)	43.94^a^ (6.26)	42.21^ab,AB^ (8.04)	34.70^b,AB^ (5.72)	**p** **=** **0.010^∗^**
NPHAp	83.92^B^ (36.90)	70.99 (31.45)	70.40^A^ (26.62)	53.52^A^ (30.52)	*p* = 0.173
BG	66.86^a,AB^ (23.68)	53.63 (18.22)^ab^	39.27^ab,B^ (14.80)	31.43^b,B^ (12.57)	**p** **=** **0.002^∗^**
	**p** **=** **0.004^∗^**	*p* = 0.051	**p** **=** **0.006^∗^**	**p** **=** **0.048^∗^**	

	After brushing				
Control	48.85 (7.62)	50.104 (10.22)	40.28 (9.54)	50.96^A^ (12.98)	*p* = 0.138
NPHAp	51.97 (22.34)	52.58 (17.69)	56.08 (24.54)	41.19^AB^ (11.04)	*p* = 0.249
BG	80.99^a^ (37.97)	57.09^ab^ (22.70)	49.46^ab^ (15.40)	37.15^b,B^ (9.85)	**p** **=** **0.009^∗^**
	*p* = 0.076	*p* = 0.802	*p* = 0.179	**p** **=** **0.031^∗^**	

*Note*. Averages in the columns accompanied by upper case letters and in the lines accompanied by lower case letters do not present significant difference by the Kruskal–Wallis test (*p* > 0.05) and Dunn post test (*p* > 0.05). **^∗^**Source: own elaboration.

## Data Availability

All data generated or analyzed during this study are included in this article. The first author, Rafael Amorim Martins, can be contacted to provide other information (address: 1680 Humaíta Street, Araraquara, SP; telephone number: +55 (16) 997625256; fax number: +55 (16) 3301-6329; rafael.a.martins@unesp.br).
